# Novel field-based approaches reveal wheat genotypic differences in nitrogen use efficiency and grain protein dynamics

**DOI:** 10.1038/s44264-026-00168-3

**Published:** 2026-06-26

**Authors:** Stéphanie M. Swarbreck, Alek Ligeza, Susie Roques, Daniel Kindred, Roger Sylvester-Bradley, Howard Griffiths, Alison R. Bentley

**Affiliations:** 1 Department of Plant Sciences, University of Cambridge, Cambridge, UK; 2 Niab, Park Farm, Villa Road, Histon, Cambridge, UK; 3 PGRO, The Research Station, Great North Road, Thornhaugh, Peterborough, UK; 4 ADAS, Battlegate Road, Boxworth, Cambridge, UK; 5 ARC Agronomic Sciences Ltd, Woodbridge, Suffolk, UK; 6 CSIRO Agriculture & Food, Black Mountain, Canberra, ACT Australia

**Keywords:** Genetics, Plant sciences

## Abstract

Achieving high yield and grain quality in wheat typically requires substantial nitrogen (N) fertiliser application. However, given economic and environmental constraints, it is critical to understand whether growers can reduce N inputs without compromising performance, and whether existing varieties differ in their ability to cope with lower N availability. Using a novel field-based experimental platform, we assessed the performance of fifteen registered wheat varieties under six N regimes and over two seasons with contrasting weather patterns. As expected, yields and grain protein contents both increased with N application, although protein content plateaued at a higher N threshold than yield. We noted higher genotypic differences in N use efficiency (NUE; defined as yield per unit of available N) under zero- N fertiliser applications, revealing intrinsic variation in low-N resilience. N-driven yield increase was more strongly associated with spike number rather than spike weight. Two varieties selected in Denmark where tight fertiliser regulations are in place were included for comparison and could achieve high yield with contrasting strategies; one with low and the other with high spike weight. In addition, using a novel stable isotope field-based method, we could show that under higher N levels, the post-anthesis N uptake was decreased and this trait is critical to achieving positive grain protein deviation (higher increase in grain protein content than expected given its yield). Our findings highlight the necessity of evaluating commercial and pre-breeding wheat germplasm under reduced N conditions to identify genotypes suited to sustainable, lower-input agricultural systems in a changing climate.

## Introduction

Nitrogen (N) is an essential macronutrient for plant growth and tends to be limiting plant primary productivity in all ecosystems except deserts^1^. The widespread availability of synthetic N fertiliser, made possible through the development of the Haber-Bosch process, has sustained crop yields in many production regions across the globe over the last century^2^. This industrial and chemical innovation and accompanying innovative agronomic practises, have been particularly important for cereal crops such as wheat (*Triticum aestivum* L.), which has a high N requirement. Concurrent advances in plant breeding led to the selection of short-strawed cultivars which were tolerant to lodging (physical displacement of stems) and more likely to allocate biomass towards the grain. Since then, wheat varieties have tended to be selected under high N inputs, which has driven increases in yield. In the UK, these have plateaued since the 1990s^3^ (FAOSTAT, 2025). Agronomic N use efficiency (NUE), the ratio of yield produced per unit of available N, is often used to describe the impact of additional N fertilisation on yield. However, the usefulness of the NUE term has been questioned^4^, since under recommended N application levels, NUE continues to remain quite low^5^, and is high under lower N availabilities (which also limit yield). Also, NUE does not account for the economic cost of increasing N applications, relative to yield, and it has recently been suggested that research should focus on N responsiveness: maximising yield relative to reduced N inputs^4^.

Soil available N is mostly taken up in the form of nitrate by wheat grown in temperate climates since it is the most available N source under those conditions^6^. Assessing soil N availability to ensure optimal supply to the crop is difficult because of seasonal fluctuations due to soil microbial activity, leaching and denitrification. However, it is essential that growers adjust their fertiliser application rate while taking into account available N. The UK RB209 Nutrient Management Guide issued by the Agricultural and Horticultural Developmental Board (AHDB) in the UK provides information on how to estimate soil available N based on soil type and previous cropping regimes. Applications of higher levels of N fertiliser are often used to maximise yield, and in some cases to increase grain protein content. In the UK, wheat grown for bread making (belonging to the UK Flour Millers Group 1) tends to require higher N applications to achieve the requisite 13% grain protein content (GPC) which is accompanied by a price premium^7,8^. In order to reduce N inputs and losses due to run-off and volatilisation, studies are required to determine whether commercially available varieties could maintain yield quantity and quality at lower applied N levels^4^. Tools are available to growers to adjust N application during the season such as measurements using NDVI (normalised difference vegetation index), or low-cost chlorophyll metres. In addition, the leaf colour chart which was developed initially at the International Rice Research Institute (IRRI) has been applied to in-season N recommendations for rice cultivation in India^9^ and adapted for wheat^10,11^. Although these tools mostly rely on assessing proxies for leaf N content, there is evidence that wheat leaf area index also increases under greater N supply^12^. This is perhaps more difficult to assess.

In Northwest Europe (especially France and the UK), many studies have evaluated the performance of winter wheat varieties under contrasting N levels over the past 5 decades. Some studies report differences amongst commercial varieties^13,14^ though varieties tend to respond more similarly^15,16^. Recently released elite genotypes tend to be less efficient in acquiring soil N in the absence of supplementation from N fertiliser^17^ and may be better adapted to acquire N dispensed at specific stages in large doses and perhaps less dependent on microbial activities. For UKFM group 1 varieties, the demand for high GPC adds to the N requirement although post-anthesis N uptake can lead to higher GPC^18^, providing a specific timepoint for intervention.

In Europe, a total of 225 winter wheat varieties released between 1969 and 2010 (mostly released between 1985 and 2010) were tested under two N rates in four experiments^19^. This uncovered significant Genotype (G) x N rate interactions for grain yield, GPC and NUE. The year of registration had a significant effect on G x N rate interaction for yield and NUE. Modern varieties had a G x N rate interaction that increased yield under high N, with a corresponding decrease under low N. These G x N rate interactions could be explained by variations in quality classes (more recent varieties tended to be higher yielding but had lower GPC and earlier flowering times). In a follow up study, tolerance indices were defined and used to identify specific QTL regions underpinning tolerance to low N^20^. Overall, reports of varietal differences in yield under varied N levels have been noted. Farmers cannot simply assume that the performance of a wheat variety will be maintained at lower N and understanding the biological basis for these differences can inform selection of cultivars better suited to low input agriculture, and reduce N losses and emissions from more intensive systems or late fertiliser applications^4^. Additionally, accounting for changing climatic conditions (winter flooding or low water recharge, summer drought) requires further research to inform farmers on timing for optimal N fertilisation schedules.

The aim of this study was to investigate the responses of modern elite wheat varieties, released between 1989 and 2014, selected across the 4 UK Flour Miller groups under contrasting N rates. The set includes varieties issued from Danish breeding programmes, which have been conducted under lower N availability compared to the UK. The use of a novel Opti-plot design allowed 6 contrasting N concentrations to be delivered to a specific variety within a single plot, across a replicated field experiment, with a modified combined harvester quantifying yields for each variety at each N rate. The field trial was repeated over two contrasting growing seasons, 2016–2018 with measurements of canopy development, chlorophyll content and soil N content, as well as ^15^N uptake and utilisation post-anthesis.

## Results

### All tested winter wheat varieties respond to increased N availability

**Fig. 1:**
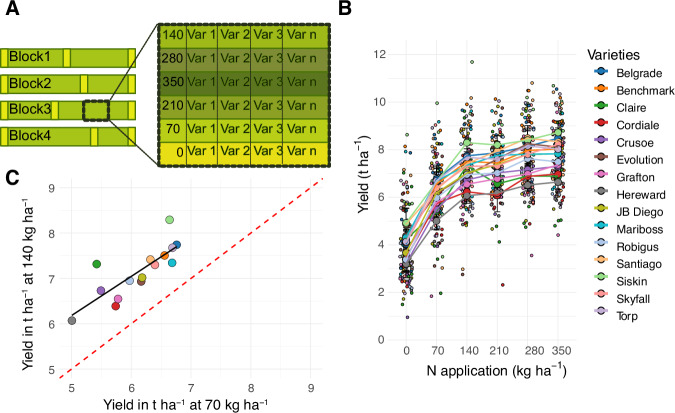
Winter wheat yield nitrogen response plateau at 140 kg N ha^−1^. **A** Opti-plot field trials were conducted where each variety was grown under 6 N levels (0, 70, 140, 210, 280 and 350 kg N ha^−1^) each within a plot and in 4 blocks. All varieties were replicated in each block. Untreated plots were positioned in the middle and at the end of each block. A total of 52 winter wheat genotypes were tested and we present here the data from the 15 commercial varieties included. **B** wheat yield (t ha^−1^) data shown as the mean ± se combined both 2017 and 2018 harvest, individual plot datapoints are also shown. **C** BLUEs for yield under 140 kg N ha^−1^ were plotted against BLUEs for yield under 70 kg N ha^−1^. Black line indicates the linear regression for the data while the dashed red line corresponds to y = x regression line. Two outlier varieties can be noted: Siskin and Claire.

A total of 15 wheat commercial varieties were grown, at six N levels (0, 70, 140, 210, 280 and 350 kg ha^−1^) in an opti-plot field trial system (Fig. 1A) over two seasons (2016–2017, 2017–2018), with contrasting weather conditions (Fig. S1). All varieties showed a typical response to the provision of N fertiliser, with an initial clear increase in yield from 0 to 70 or 140 kg N ha^−1^ (Table S2, Fig. 1B). For most varieties, the yield tended to plateau above 140 kg N ha^−1^, except for Cordiale which plateaued at 70 kg N ha^−1^. The yield achieved in harvest year 2018 tended to be lower (reaching a maximum of 8.8 t ha^−1^) than that achieved in harvest year 2017 (reaching, a maximum of 11.7 t ha^-1^, Fig. S2). While all varieties responded to the provision of N fertiliser, there were significant difference in yield amongst varieties at different N levels (Fig. S3A). Under no additional N supply, Siskin and Santiago showed significantly higher yield compared to Cordiale, Robigus, Crusoe and Hereward. Siskin remained a high yielding variety maintained at high N availability. Danish derived varieties, Belgrade, Torp and Mariboss were the top performers at low (70 kg N ha^−1^) N level. While there was overall good correlation between performance at 70 kg N ha^−1^ and 140 kg N ha^−1^, the varieties Claire and Siskin produced significantly higher yields under 140 kg N ha^−1^ than predicted for their performance at 70 kg N ha^−1^ yields (Fig. 1C). These appear as clear outliers relative to the regression line, which reflects a higher N responsiveness than observed for the remaining varieties.

### NUE decreases with increasing N application rate

**Fig. 2:**
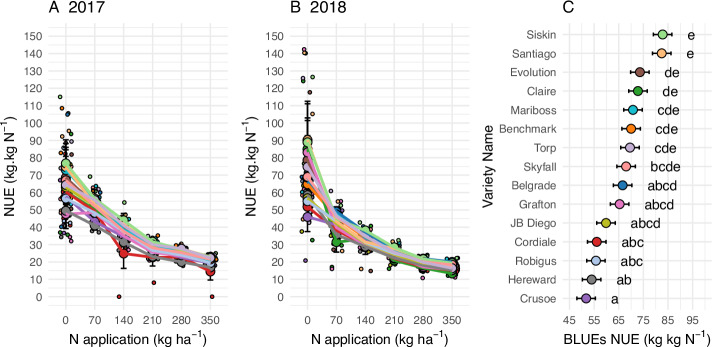
NUE decreases with increasing N application. **A**, **B** NUE calculated as the yield per unit of N available (sum of soil available N and fertiliser applied) for each variety measured at each of the six N application rates. Data shown as individual datapoint and the mean ± se separately for 2017 and 2018. **C** Data shown as BLUEs (Best Linear Unbiased Estimators) from combined data for 2017 and 2018 under 0 N application. Letters indicate significant differences amongst varieties.

NUE was calculated as the ratio of yield per unit of available N (i.e. estimated as the sum of residual soil N and applied N). Overall, there was no significant difference between 2017 and 2018 (Fig. 2, lmer n.s.). The differences amongst varieties were significant at the lowest N rate (lmer, *p* < 0.01), and there was a significant interaction between variety and N treatment (lmer, *p* < 0.05). Santiago and Siskin showed the highest NUE, while Crusoe showed the lowest (Fig. 2). NUE decreased with increased N availability (lmer, *p* < 0.01). NUpE (defined as the ratio of total N uptake per unit of N available) also declined with the addition of N fertiliser (lmer, *p* < 0.01) and no significant differences amongst varieties could be measured (Fig. S4A) while the Nitrogen Harvest Index (proportion of N in the grain to the total N taken up) remained similar between 0 and 210 kg N ha^−1^ treatments (Fig. S4B, lmer, *n.s*.).

Grain yield increased with the date of variety registration with more recent varieties showing higher yield overall (Fig. S5). However, in the absence of N fertiliser, the slope was lower (0.04 t ha^−1^ per year, compared to 0.06 t ha^−1^ per year under 70 kg N ha^−1^) and the correlation was not significant (*p* = 0.07), compared to the other N application rates. With applied N rates above 210 kg N ha^−1^, the correlation tended to be greater than 0.6.

### NDVI is an indicator of N dependent yield increase

**Fig. 3:**
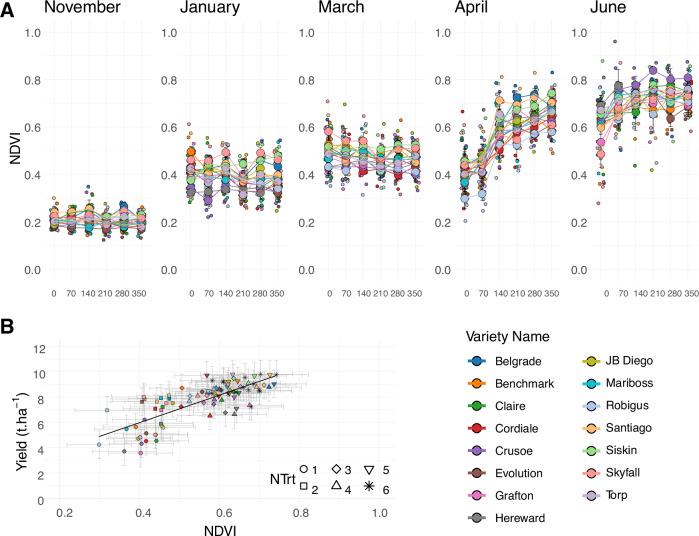
NDVI measurements at GS31 are correlated to yield. **A** NDVI measurements were conducted on each plot at five different timepoints during the season- (November- GS13, January- GS23, March- GS25, April- GS31, and June- GS61). Data shown as individual plot datapoints and mean ± SE per variety. **B** NDVI measurements at GS31 are positively correlated with yield. Data shown as individual plot datapoints and mean ± SE per variety.

NDVI (Normalised Difference Vegetation Index) measurements were conducted at five timepoints throughout the first trial season (November- GS13, January- GS23, March- GS25, April- GS31, and June- GS61) and identified significant differences between varieties at all time points except late in the season (June). NDVI values increased throughout the season indicating increased ground coverage (Fig. 3A). In April, a significant response to an increase in N availability was detected from 70 to 140 kg N ha^−1^ (Fig. 3A). At this timepoint, there was a significant correlation between NDVI measurements and yield (Fig. 3B, R^2^ = 0.48, *p* < 0.01). Overall, there was no significant interaction between varieties and N treatment. While most varieties showed a positive correlation between yield and NDVI measured in April, this was not the case for Cordiale (Fig. S6). It is worth noting that the correlation between yield and NDVI was strongest under lower N levels (Fig. S7).

Leaf greenness was measured using SPAD at flowering in both 2017 and 2018, and clearly increased with additional N availability (Fig. S8). The leaf colour chart (LCC) was also used in 2018 to capture visible changes in leaf greenness (Fig. S8). Overall, there was good correlation between SPAD and LCC measurements (*R*^*2*^ = 0.9, *p* < 0.01).

### Nitrogen dependent yield increase is linked to increased shoot number when rainfall is sufficient

**Fig. 4:**
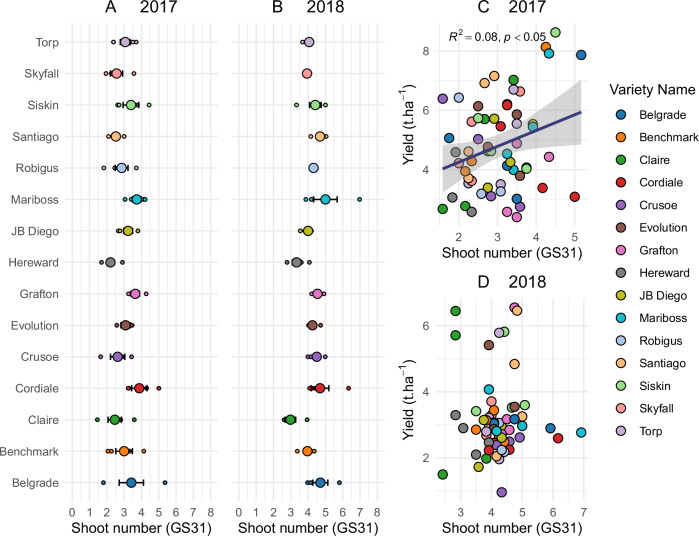
Varieties show different shoot number which is correlated with yield only in 2017. **A** 2017**, B** 2018 Shoot number per plant at GS31. Data shown as individual plant datapoints and mean ± SE. **C** 2017, **D** 2018 Shoot number at GS31 was positively correlated with yield only in 2017, not in the year of the late drought (2018). Data shown as individual datapoints.

Wheat yield is the product of grain number (grain number per spike and spike number) and grain weight. The number of shoots was higher in 2018 (ranging from a mean of 2.98 shoots per plant for Claire to a mean of 5 shoots per plant for Mariboss), compared to 2017 (mean of 2.21 shoots per plant for Hereward to a mean of 3.88 shoots per plant for Cordiale, Fig. 4). There were significant differences in shoot number amongst varieties in both years (lmer, *p* < 0.05) and differences amongst varieties. These were generally higher in the wetter winter of 2018 (Fig. S1), with Hereward and Claire showing a lower shoot number compared to other varieties (Fig. 4). There was a positive, though weak, correlation between shoot number at GS31 and yield but only in the harvest year 2017 (R^2^ = 0.08, *p* < 0.05) and not in harvest year 2018 (*n.s*.), when there was a late drought following anthesis (Fig. S1).

### Spike weight does not respond to increased nitrogen availability

**Fig. 5:**
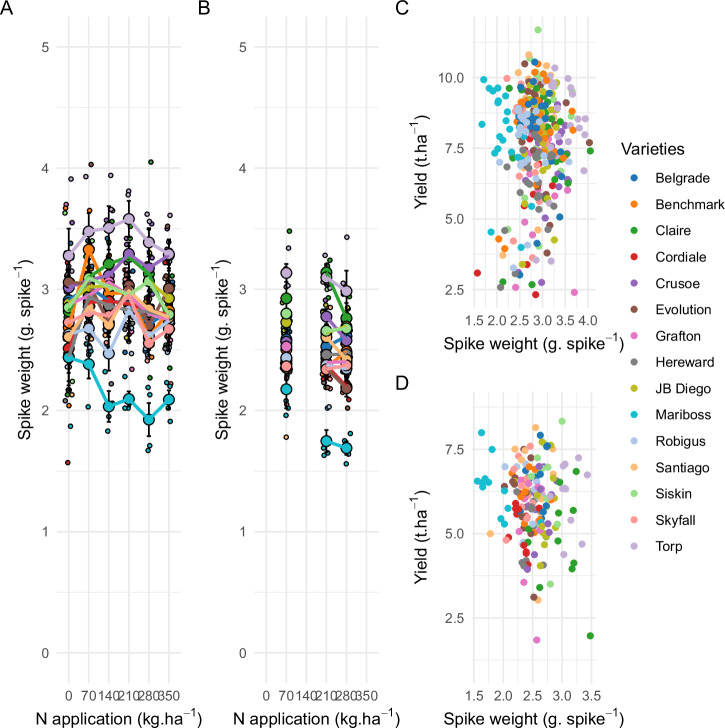
Wheat spike weight does not respond to increased nitrogen availability. **A**: 2017, **B**: 2018 Spike weight measured before harvest. Data shown as individual plant datapoints and mean ± SE. **C**: 2017, **D**: 2018. There is overall no correlation between spike weight and yield. Data shown as individual datapoints.

Spike weight was not significantly affected by N level (lmer, n.s.) in both 2017 and 2018 (Fig. 5). There were significant differences amongst varieties in both years (Fig. 5). While Mariboss generally showed lower spike weight compared to all other varieties (lmer, *p* < 0.01), Torp showed significantly higher spike weight (Fig. S9). The spike weight for Crusoe was also significantly higher compared to other varieties (lmer, *p* < 0.01).

### Wheat grain protein content plateaus at higher N availability than yield

**Fig. 6:**
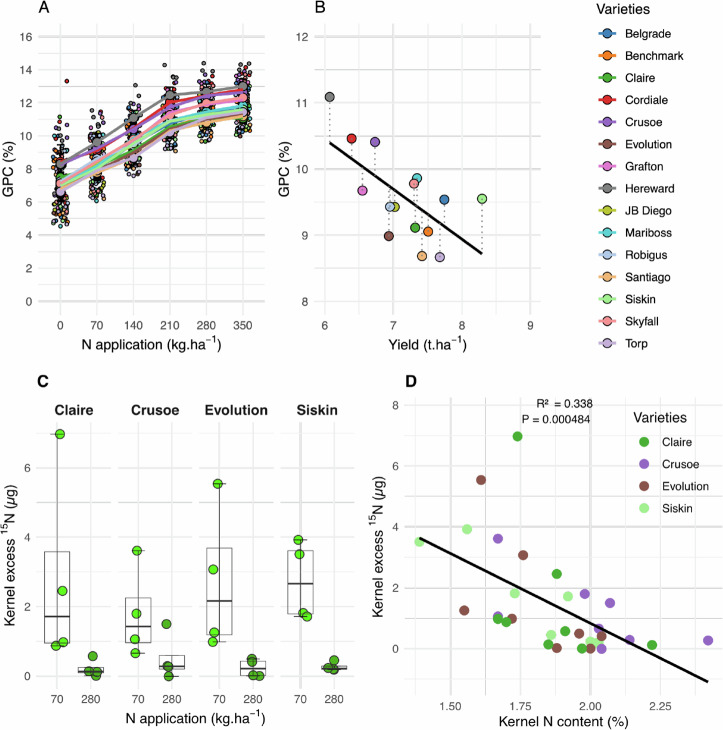
Wheat grain protein contents plateau at higher N rate than yield. **A** Wheat grain protein content (%) data shown as the mean ± se combined both 2017 and 2018 harvest, individual plot datapoint are also shown. **B** Grain protein content is negatively correlated with yield. Data are shown as BLUEs at 140 kg N ha^−1^. **C** Kernel ^15^N excess, as indicator of post-anthesis nitrogen uptake (PANU) measured for four wheat varieties under two N application levels in 2018. Data shown as mean ± se for one plant per plot and for each of the four replicates plots. **D** The kernel ^15^N excess is negatively correlated with the kernel N content at the time of measurements. Data shown as individual data point and linear model for the regression.

All varieties showed a clear response to the provision of N fertiliser, with an increase in GPC (Fig. 6A). For most varieties, GPC tended to plateau at 210 kg N ha^−1^, which is a higher N level than the yield plateau. The overall GPC achieved in 2017 tended to be higher (range of 7.19–14.39%) than that achieved in 2018 (range of 4.54–12.15, Fig. S2), again showing the impact of the late drought. While the N dependent yield and GPC increase showed a positive correlation (2017: R^2^ = 0.26, *p* < 0.01; 2018 R^2^ = 0.43, *p* < 0.01) overall, at each N level we observe the expected negative correlation between yield and GPC (Fig. S11).

### Positive grain protein deviation is linked to post-anthesis N uptake

There was significant differences grain protein content amongst varieties (Fig. S10) as expected given their UKFM group. Some varieties showed positive grain protein deviation (GPD, representing an increase in grain protein content relative to yield, and hence positioned above the regression line in Fig. 6B) including Siskin (also higher yielding) and Crusoe (lower yielding), while others showed a negative GPD (Claire and Evolution, Fig. 6B). Positive GPD has been linked to increased post-anthesis N uptake (PANU)^18^, and this was measured in the field using ^15^N labelling (Fig. 6C). PANU is defined as the plant capacity to take up N directly from the soil solution after anthesis, and using it as a source of N for the developing grain rather than relying entirely on remobilised N from senescing leaves. While there were no significant differences amongst varieties, there was increased post-anthesis N uptake under lower N supply (70 kg N ha^−1^) compared to higher N supply (280 kg N ha^−1^, lmer *p* < 0.01). A negative correlation between kernel N content and kernel ^15^N excess was measured in the field, suggesting higher PANU potential in lower N-containing grains.

**Fig. 7:**
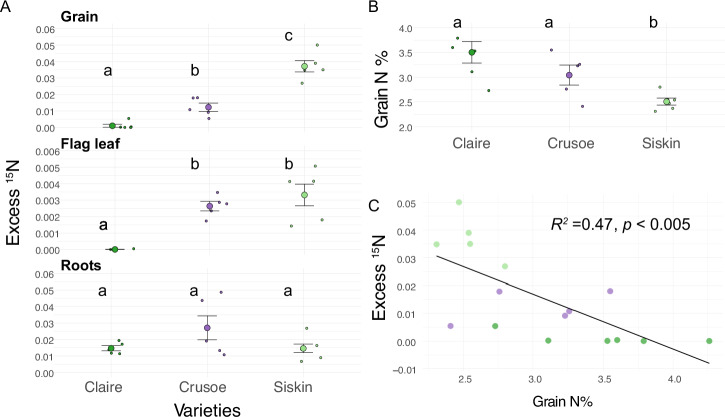
Pot based post-anthesis N uptake measurements. **A** ^15^N excess was measured in the kernel, flag leaves and roots of wheat plants grown under controlled conditions and exposed to ^15^N labelled ammonium nitrate. **B** Kernel N content. **C** The kernel ^15^N excess is negatively correlated with the kernel N content at the time of measurements. Data shown as individual data point for 5 biological replicates and linear model for the regression.

In a separate experiment, PANU was measured in plants grown in pots under controlled conditions following a similar method of application of ^15^NH_4_^15^NO_3_ to the growth substrate. Under these conditions, roots could be washed and analysed as well as the flag leaves and grains. Grains represented the largest store of ^15^N post-anthesis while very little ^15^N remained in the flag leaf (Fig. 7A). Under these conditions, a significant difference was recorded, with positive GPD varieties (Crusoe and Siskin) showing higher PANU compared to Claire which has negative GPD (Fig. 7A). Overall, there was also a negative correlation between grain N content at the time of analysis and PANU as measured through ^15^N excess (Fig. 7C), consistent with the field data.

## Discussion

Balancing the need for wheat production for food and feed with environmental impact associated with modern fertiliser inputs is a challenge that requires continued investigation, especially in the context of seasonal variations in water supply and drought associated with climate change. Here we report the response of elite winter wheat cultivars from Northern European breeding programmes (all released in the last 25 years) to N rates ranging from 0 to 350 kg N ha^−1^. There was a clear positive effect of N application on the yield of all cultivars tested, while some varietal differences could be measured. NUE showed a strong decline under even modest N application for all varieties. We show that the N-dependent yield increase is linked to an increased spike number, rather than spike weight, thus identifying a possible mechanism for N responsive yield increase and a target for crop breeders.

Field trials across the broad range of N application rates used here are unusual because of the technical complexity. Unless wheat is grown under organic farming practices^21^, some level of N is added which makes the level 0 in our study unique. At the other end of the spectrum, applying 350 kg N ha^−1^ is not economically viable for farmers (except where fertiliser is heavily subsidised) and results in environmental losses. However, assessing diverse wheat cultivars under this range of N applications rate is valuable as it allows precise quantification of their N responses. The opti-plot testing approach used here allows for testing of 6 N levels with sufficient plot sizes to allow for reliable agronomic assessment of crop growth and grain yield. The opti-plot design uses a criss-cross design in which discrete mainplots (here varieties) are crossed with dose treatment subplots (here N rates), and uses smaller than standard plots to enable experimentation with limited seed supplies. The plots and treatment are arranged to ensure some level of randomisation and reduces the likelihood of impact from high N sub-plots onto low N sub-plots. Yield of all varieties tested tended to plateau at 140 kg N ha^−1^, in both 2017 and 2018 even though climatic conditions (late drought) which probably limited yield in 2018.

All varieties tested here showed a clear increase in yield under higher N availability. Since the varieties had all been selected for and commercialised in the last 25 year, this is expected. Modern wheat cultivars tend to show higher yield responses under high N availability compared to older (and taller) varieties^22^. We also noted increases in yield at all N levels related to the year of registration which is in-line with similar analyses conducted on German wheat germplasm^5^. The positive correlation between year of registration and yield was more apparent under higher N rate (Fig. S5). For example, the correlation between yield and year of registration was not significant under no N supply and the slope was 0.04. However, under N rates greater than 210 N kg ha^−1^ the correlation was significant and the slope was greater at 0.07. This suggests that perhaps selecting under lower N levels would lead to greater genetic gains for performance under lower N. While all varieties tended to respond to the same N rate, selected varieties (Siskin and Claire, Fig. 1C) showed a greater than expected N responsiveness suggesting that this is a trait that has not yet been maximised for all commercially released varieties. N responsiveness is the capacity of plants to respond to increased N availability. We argued that varieties showing higher N responsiveness should be a selection marker, as this trait is linked to the molecular signalling associated with plant N uptake and mobilisation^4^. The response for Cordiale to higher N was lower, and we propose this to be linked to its early flowering phenology (Fig. S12), which may have resulted in mis-timed N fertiliser application. It is worth noting that Danish varieties performed well under 70 kg N ha^−1^, though their overall NUE was not particularly improved compared to varieties selected through breeding programmes in the UK (Fig. 2C). However, they are able to achieve high yields using different strategies as Mariboss showed a very low spike weight that was compensated by greater spike numbers. It is unclear whether this is due to the climatic or edaphic differences between UK and Denmark. Nevertheless, they can achieve high yields using different strategies as Mariboss showed a very low spike weight that was compensated by greater spike numbers. Overall, this also suggest that multiple complementary approaches are needed to select varieties that can perform well under a range of N rate.

Although NUE does not effectively capture the economic costs associated with N application (which makes N responsiveness such a useful metric), we confirmed that it was much greater under lower N availability, in line with other studies^5^. The higher NUE under no fertiliser application can be associated with either increased NUpE (the ratio of total N uptake per unit of N available) or NUtE (yield per unit of N taken up by the plant). Higher NUpE can be achieved under no additional N application when microbial dependent N sources become more important^23^. Under no additional N application, soil processes can be improved or increase recruitment of beneficial microbes, which tend to be inhibited at elevated N availability^24^. In our study, we could see a similar pattern of increase NUpE under low N but no change in NHI (grain N content relative to above-ground plant N) suggesting that increased uptake efficiency underpins the higher NUE without any fertiliser application. This supports the finding that in a study of French wheat varieties (historical, pre-Green Revolution and more recent varieties) grown under two levels of N (no addition, and 170 kg ha^−1^ addition), NUpE accounted for more of the variation of NUE for grain yield at N0 than under N fertilisation^25^. Under low N, wheat plants can develop more extensive root systems to explore and exploit a larger soil volume and access a higher proportion of soil available N^26–28^.

It is interesting that differences in grain yield, hence NUE, amongst varieties are very strong at N0. It is often thought that plants need to be grown under optimal/high N rate to see differences amongst varieties and enable efficient breeding. However, here, the strongest differences were seen at lower N level. Perhaps it is because these varieties were selected under generally higher N levels that no differences amongst variety specific NUE can be observed at high N levels. This raises the potential of including reduced N level screening in selection of germplasm in breeding and assessment of commercial varieties in the UK^15^ as is done in other countries such as France.

Yield is a complex trait that includes grain weight and grain number (which itself is dependent on spike number and number of grains per spike). Here we show that spike number varies amongst varieties and is correlated with grain yield, although only under sufficient moisture (2017). Our data show a similar pattern to data from foxtail millet^29^ where N-dependent yield increased is underpinned by increases in grain number rather than grain weight. This lack of increase in grain weight under increasing N level is also noted as spike weight remained stable at all six N levels in both trial years of the experiments. It remains to be seen whether improving individual grain weight response to increased N may lead to further yield increase under higher N availability. Danish varieties Torp and Mariboss adopted contrasting strategies to achieve high yield. Torp showing higher spike weight compared to Mariboss under all N levels, and lower spike numbers.

Relying mostly on increased tillering capacity to drive yield may also be a risky strategy in a changing climate. Although here we only have one year worth of data that includes a drought (2018), the lack of positive correlation between grain yield and shoot number in this year is interesting. Shoot numbers were also greater in 2018 compared to 2017 (Fig. 4) and it is not clear whether a positive correlation would have been found should these have remained on par with shoot numbers in 2017.

In our study, N dependent GPC tended to plateau at higher N application rates than yield. GPC of varieties was also negatively correlated with their yield. However, when yield increase is driven by increasing N, there is an overall positive correlation between yield and GPC. It is only at each N level, that a negative varietal correlation was noted. From this analysis, we could identify varieties with higher yield such as Evolution and Siskin, and varieties with lower yield such as Claire and Crusoe. In terms of grain protein content relative to yield (GPD), both Crusoe and Siskin showed positive GPD while Claire and Evolution showed negative GPD. Such data provides a basis for crop breeders to select varieties with enhanced yields in association with more sustainable N inputs.

Late N applications are often used to enhance GPC and nudge grain quality towards UKFM bread-making requirements. Using an ^15^N based method we were able to measure post anthesis N uptake (PANU) at a specific time point and showed overall a lower PANU (expressed as ^15^N kernel excess) at higher N rates. This is a novel method for measuring PANU, as previous reports included estimating the total N content in the grain and straw at maturity in comparisons with the total plant N at anthesis^30^. Our ^15^N based measurement is useful as it allows temporal resolution of PANU. This trait remains quite poorly understood and somewhat goes again the general concept that N in the grain is derived from the remobilisation from vegetative tissues. However, it was shown to be particularly relevant to the improvement of GPC^18^. While no varietal differences could be noted in the field, under controlled conditions (well-watered) there was significant differences, with varieties showing positive GPD also showing higher PANU. Our findings that under higher N availability PANU declines are in agreement with a previous report^31^, which also suggested a key role for the high affinity nitrate transporter *TaNRT2.1*. There is a need for further exploration of the genetic basis for PANU, especially in terms of regulation by plant-N status and contribution from remobilisation of existing N assimilates. Our data suggest that grain N content itself is correlated with PANU but the nature of the signalling mechanism from grain N status to root uptake is not known currently. Although there are pathways which signal from shoot to root including the carboxy-terminally encoded peptide downstream pathways, or phytohormones based systems (e.g. auxins or cytokinins; for review^32^), it is unclear whether these are involved in the signalling from the developing grain to the roots.

From an agronomic and crop breeding perspective, the novel opti-plot approach, combining a wide range of N availabilities across two contrasting years in the field has provided insights for the traits that underpin higher performance in these conditions. Given the low level of genetic diversity amongst commercial wheat varieties in the UK, evaluating these under an extensive range of N level enables resolution of their N response differences, with a view to the selection of varieties which sustain yield and grain quality at lower N inputs. Our work also demonstrated that measurements of NDVI (and other low technology solutions, such as the LCC) throughout the season, and especially at GS31, provided a good indication of overall yield. However, climatic conditions in both years contributed to overall yield and grain quality, whether in terms of lower tillering in a dry winter (2017) or a reduction in spike productivity in a late season drought. These findings are a step towards providing farmers with a clearer understanding of how changing climatic conditions can alter the effectiveness of late N applications, whether in terms of enhancing GPD or recovering the financial cost of the additional inputs. In future, such an economic N optimum will need to be associated with individual crop varieties, and more sustainable farming practices, which minimise N losses and maximise grain yield and quality.

## Methods

### Opti-plot field trials

A total of 15 winter wheat commercial varieties were selected for field testing, representing UK commercially available lines from each of the four UK Flour Millers groups as well as varieties selected for the Danish market, where restrictions on N applications are in place^33^ (Table 1).

**Table 1:** List of winter wheat varieties included

Varieties	UK/Denmark	UKFM Group^a^	Year of registration^b^
Claire	UK	3	1997
Cordiale	UK	2	2002
Crusoe	UK	1	2010
Evolution	UK	4	2012
Grafton	UK	4	2007
Hereward	UK	1	1989
JB Diego	UK	4	2006
Robigus	UK	3	2001
Santiago	UK	4	2009
Siskin	UK	2	2014
Skyfall	UK	1	2012
Belgrade	Denmark		2014
Benchmark	Denmark		2014
Mariboss	Denmark		2008
Torp	Denmark		2013

^a^UK Flour Miller Groups provide information on the suitability of the grain produced for downstream use. For example, Group 1 varieties grains must be of consistent milling and baking performance, while Group 4 varieties are mainly grown for animal feed.

^b^Year of registration indicates year of completion of the statutory National Listing.

Two field experiments were conducted in Stetchworth (Suffolk, UK) on different fields on the same farm in 2016/2017 (52°2180’ N, 0°3707’ E) and 2017/2018 (52°2125’ N, 0°3721’ E). The trial was drilled on 28th September 2016 and on 5th October 2017. Soil types were sandy loams. Climatic conditions throughout the growth period, including rainfall and temperature patterns, are presented in Fig. S1. The total rainfall from 1st January until the 31st August was lower in 2017 (215.8 mm) compared to 2018 (387.4 mm). However, during the grain filling period post-anthesis (1st June–15th August), the total amount of rainfall was much higher in 2017 (126.2 mm) compared to 2018 (54.8 mm). Trials in both years used the experimental field design (Fig. 1A), that held four blocks, each containing variety mainplots of 2 m × 32 m each, plus 2 m × 32 m ‘uniformity plots’ at each end and in the centre of each block, flanked by 2 m × 32 m discard plots. Varieties were grouped and the groups were randomised in each block. N treatments ran across blocks in 4 m strips, so that each 32 m plot is split into eight 4 m subplots. The central six N subplots included six N treatment (0, 70, 140, 210, 280 and 350 kg N ha^−1^) achieved through the application of ammonium nitrate granules (Fig. 1A), the subplot at each end of the plot were treated at 210 kg N ha^−1^ rate and discarded because it contained a tractor wheeling. The order of N treatments in each block was randomised, with the randomisation restricted such that adjacent N treatments did not differ by more than 2 N levels. All plots were planted at a seed rate of 350 seeds m^−2^ and replicated in each block. A total of 52 winter wheat genotypes including 15 commercial varieties were tested and we present here the data from the commercial varieties only (Table 1).

In the first trial year, soil mineral N was measured on 26th January 2017 to be 75 kg N ha^−1^ (soil analysis was completed at a commercial lab following BS EN 13652:200). Polysulphate was applied at a rate of 101.5 kg ha^−1^ across the trial on the 10/03/17 to avoid S limitation (48.7 kg ha^−1^ SO_3_, no N). In the second year, soil mineral content was measured on 30th January 2018 to be 25 kg N ha^−1^. A rate of 100 kg ha^−1^ ammonium sulphate was applied across the trial 20/03/18 to avoid S limitation (21 kg N ha^−1^ N, 60 kg ha^−1^ SO_3_). In each season, N was applied in three splits (details of the dates and N applied at each split is shown in Table S1). Field trials were managed following typical practices including application of fungicides, herbicides and pesticides. Grain yield for each individual N application level was measured by plot harvester (Sampo 2010) and expressed at 85% dry matter. Grain protein and moisture contents were measured using Near-Infrared Spectroscopy at a commercial lab.

### NDVI measurements

NDVI measurements were conducted using a RapidScan CS-45 (Holland Scientific) on 2nd November 2016, 27th January 2017, 1st March 2017, 25th April 2017 and 1st of June 2017.

### Shoot number at GS31

The number of shoots per plant was counted on ten uprooted plants from each sub-plot at GS31 (the start of stem elongation^34^).

### Pre-harvest grab samples

Pre-harvest samples of 30 whole shoots were cut at ground level from each subplot at two N rates (0 and 210 kg N ha^−1^). Samples were split into ears and straw and oven-dried at 80 °C for 24 h before weighing. The ears threshed to separate grain and chaff, and the grain re-dried and weighed. Samples of straw + chaff were analysed by Dumas for %N content. Plot yields were used to calculate grain, chaff and straw biomass (t ha^−1^) and N content (kg ha^−1^). From these data, a series of indices were calculated as follows: NUE was calculated as the ratio of yield (t ha^−1^) divided by the N available (kg ha^−1^); NUpE was calculated at the ratio of the total N uptake (as measured by N in the grain in and N in the straw or chaff in kg ha^−1^ at the end of the season) per unit of N available (kg ha^−1^); while NHI correspond to the ratio of N in the grain (kg ha^−1^) to the total N taken up (kg ha^−1^). The total N available was estimated as the sum of N applied added to the residual N level measured at the start of the season.

At total of 10 spikes was collected per plot; these were dried then weighed.

### Chlorophyll content and leaf measurements

Chlorophyll contents were estimated using a portable chlorophyll content metre (CCM-200 plus, Opti-Sciences, Hudson, USA) on 12 flag leaves from individual plants per sub-plot between 1st June 2017 and 21st June 2017 and on 8 plants per sub-plot between 5th June 2018 and 21st June 2018, around flowering time (after GS61). Assessments using the leaf color chart (LCC) were conducted over the same period in 2018^10,11^.

### Post anthesis nitrogen measurements

For a subset of varieties (Claire, Crusoe, Evolution and Siskin), post-anthesis nitrogen uptake (PANU) was estimated using a ^15^N isotopic method. In the field, a volume of 10 mL of a 1 mM ^15^NH_4_^15^NO_3_ (^15^N_2_ 98%, Cambridge Isotope Lab Inc.) solution was added to the base of one plant at milk stage (GS75) and one tiller of the targeted plant was collected after 2 days. Additional tillers from untreated plants were collected from each plot as control. Tillers were then dried at 75 °C for 48 h or until constant weight. All samples were weighed, ground to a fine powder and analysed for isotopic ratio of ^14^N/^15^N. Post-anthesis N uptake was estimated as the capacity of plants to take up ^15^N over the 2-day labelling period.

In a separate pot experiment, plants (varieties Claire, Crusoe and Siskin) were grown under controlled conditions with 16 h photoperiod, 20 °C day/18 °C night, light intensity (200 μmol m^−2^ s^−1^), in 1 L pots on a 1:1 (v/v) mixture of sand and a clay-based substrate called terragreen, supplemented with a modified Hoagland solution^31^. Nutrient solution including 1 mM KH_2_PO_4_, 1.5 mM KNO_3_, 0.25 mM Ca(NO_3_)_2_, 4H_2_O, 2 mM MgSO_4_, 7H_2_O, 3.25 mM CaCl_2_, 2H_2_O, 3.5 mM KCl, 1 mM NH_4_NO_3_, 10 μM H_3_BO_3_, 0.7 μM ZnCl_2_, 0.4 μM CuCl_2_, 2H_2_O, 4.5 μM MnCl_2_, 4H_2_O, 0.2 μM MoO_3_ and 50 μM EDFS-Fe, was supplied every 2–3 days. At GS75, a volume of 10 mL of a 1 mM ^15^NH_4_^15^NO_3_ was provided to each pot. Samples were collected 2 days post-labelling. Roots were washed from the sand and terragreen. Grains were collected from the middle of the spike from the main tiller. Flag leaves were also collected from the main tiller.

Plant material was dried at 75 °C for 48 h, before grinding to a fine powder using a Tissue Lyser (Qiagen). Samples sealed in tin capsules were analyzed for percentage carbon, percentage nitrogen, ^12^C/^13^C (δ^13^C) and ^14^N/^15^N (δ^15^N) using a Costech Elemental Analyzer attached to a Thermo DELTA V mass spectrometer in continuous flow mode. The dried sample was carefully weighed (0.5 mg) into a tin capsule, sealed and loaded into the auto-sampler (analyses conducted at the Godwin Laboratory, Department of Earth Sciences, University of Cambridge). Precision of analyses is better than 0.1‰ for ^12^C/^13^C. Data are presented as ^15^N excess, which was calculated based on measurements of δ^15^N and tissue N % as described previously^35^.

### Statistical analysis

Statistical analysis of the field trial data was conducted in R and using the lme4 package. A linear mixed model was used to analyse the data: lmer(variable ~ Year * Variety * N Treatment + (1| Year:Block) + (1| Year:Block:Plot) + (1| Year:Block:Subplot). The term “(1| Year:Block)” was included to account for the block as a random effect, while “(1| Year:Block:Plot)” was included to account for plot within blocks as random effect (as each varieties is one plot) while “(1| Year:Block:Subplot)” was included to account for the fact that all N levels are on the same strip. Best Linear Unbiased Estimates and confidence intervals were calculated using the emmeans function from the emmeans package in R.

## Supplementary information

Supplementary information

## Data Availability

Data are publicly available at 10.5281/zenodo.19593898.
