# Supplementary material for: Novel field-based approaches reveal wheat genotypic differences in nitrogen use efficiency and grain protein dynamics
Source: NPJ Sustain Agric. 2026 Jun 26;4(1):53. doi: 10.1038/s44264-026-00168-3 (PMC13309280; doi:10.1038/s44264-026-00168-3)

Supplementary figures:

Figure S1: Total rainfall and temperature in Cambridge over the duration of the two-year trial *(Blue lines, left axis: daily rainfall; Orange lines, right axis: maximum (full) and minimum (broken) daily temperatures)*.

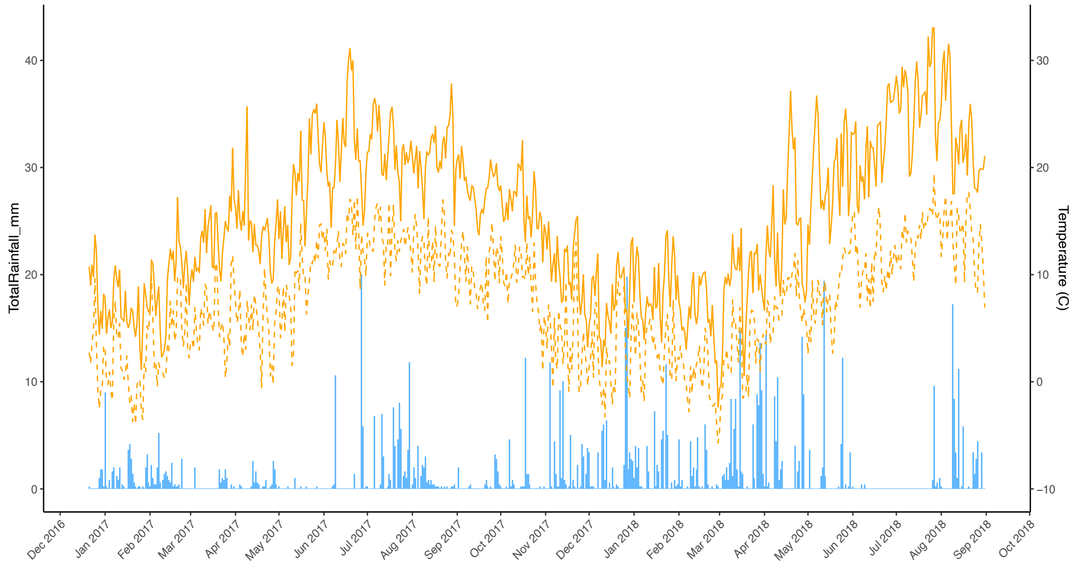

Table S1 N fertiliser application rate per split

Target Rate (kg N ha^{-1})	Actual rate (kg N ha^{-1})	Date of application for each of the three splits		
		**10/03/2017**	**29/03/2017**	**10/04/2017**
0	0	0	0	0
70	69	0	34	35
140	141	40.2	50.3	50
210	213	40.2	87.4	85
280	280	48.8	121	110
350	353	48.8	159	145

Target Rate (kg N ha^{-1})	Actual rate (kg N ha^{-1})	Date of application for each of the three splits		
		**20/03/2018**	**26/04/2018**	**09/05/2018**
0	0	0	0	0
70	69	0	34.5	34.6
140	140	39.8	50.3	50.3
210	210	39.8	85.3	85.3
280	284	60.7	111.5	111.5
350	350	60.7	144.5	144.5

Table S2 Statistical analysis for yield response (Data shown in Fig. 1)

	**P value**
Year	$p < 0.001$
Variety	$p < 0.001$
N Treatment	$p < 0.001$
Year x Variety	$p < 0.1$
Year x N Treatment	n.s.
Variety x N Treatment	n.s.
Year x Variety x N Treatment	n.s.

Figure S2: Yield and GPC were lower in 2018 compared to 2017. Wheat yield (t ha^{-1}) and grain protein data shown as the mean ± SE separately for 2017 and 2018, individual plot datapoint are also shown.

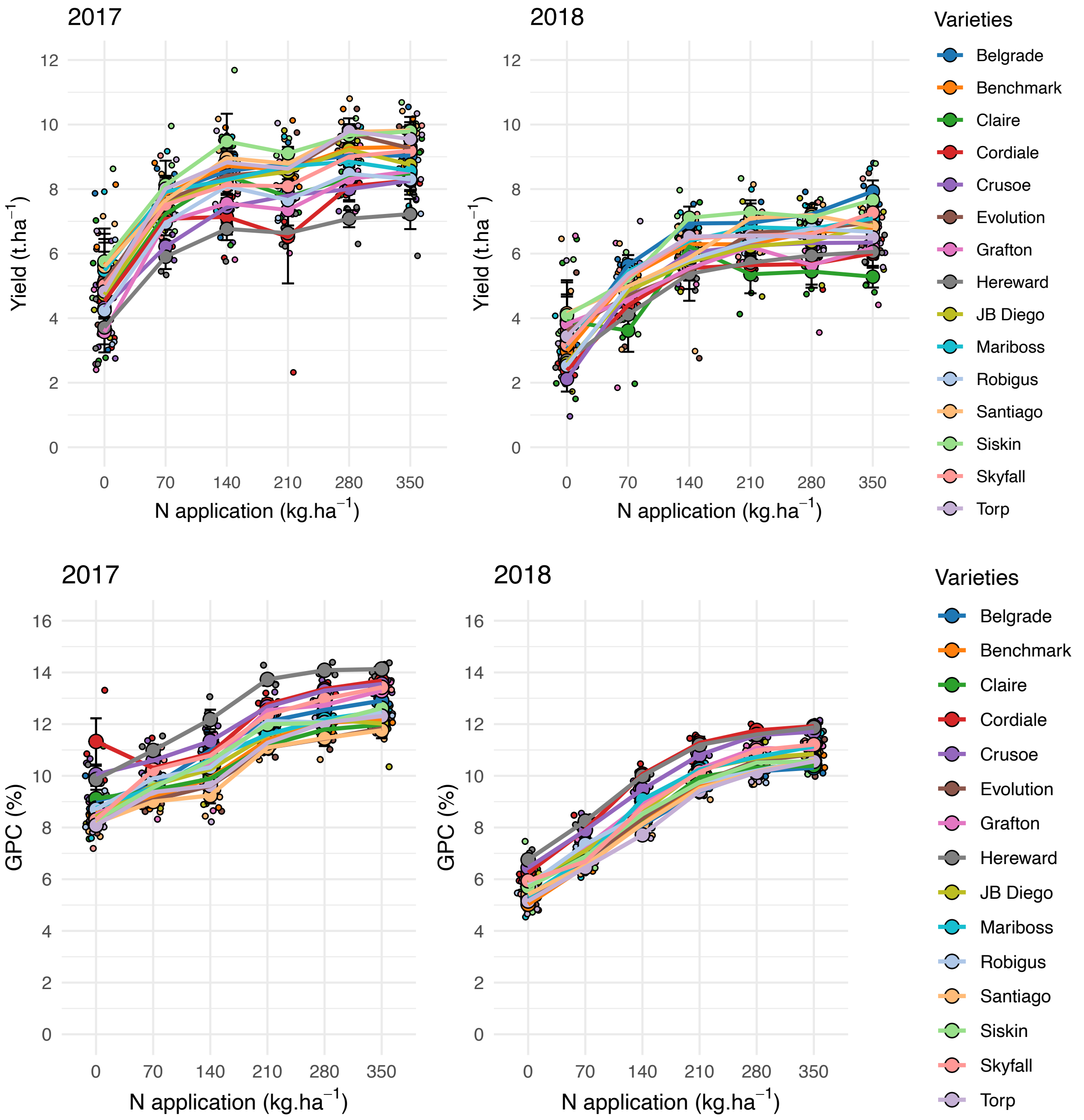

Figure S3: Varietal performance under varied N rates. BLUEs for each N level are shown for each variety. * indicates statistical significance

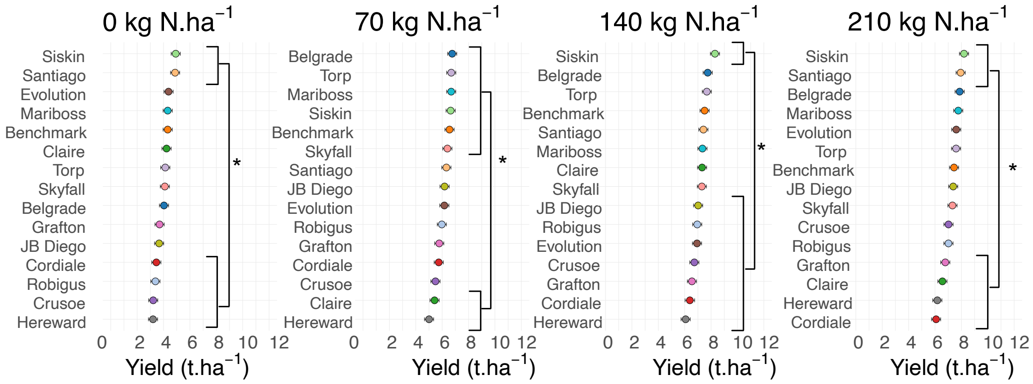

Figure S4 N Uptake Efficiency and Nitrogen Harvest Index (NHI). NHI calculated as Grain N content relative to above-ground plant N content.

A

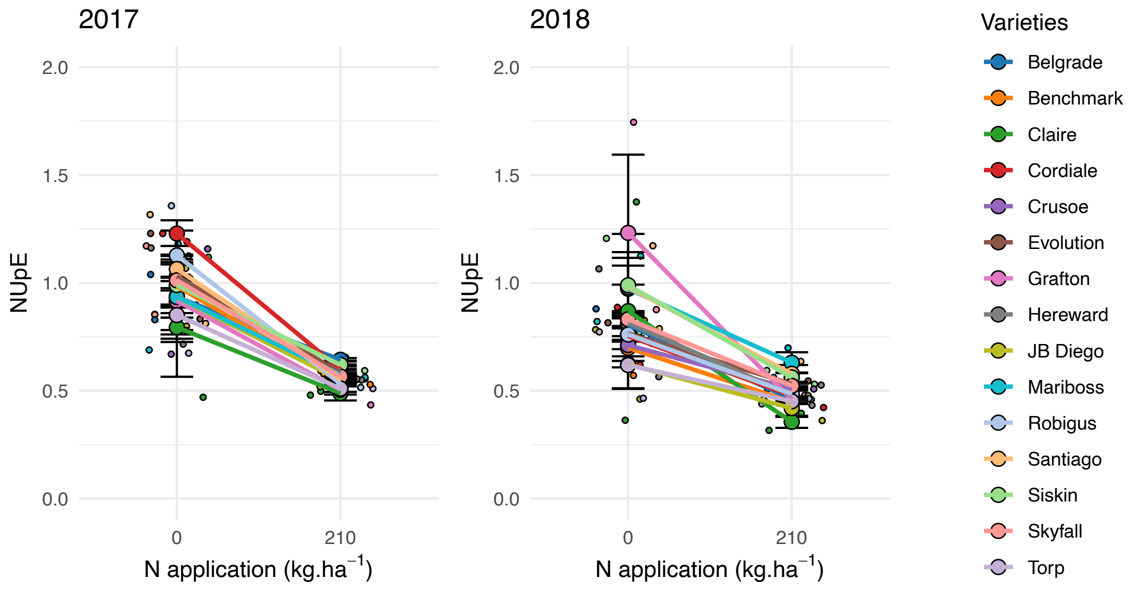

B

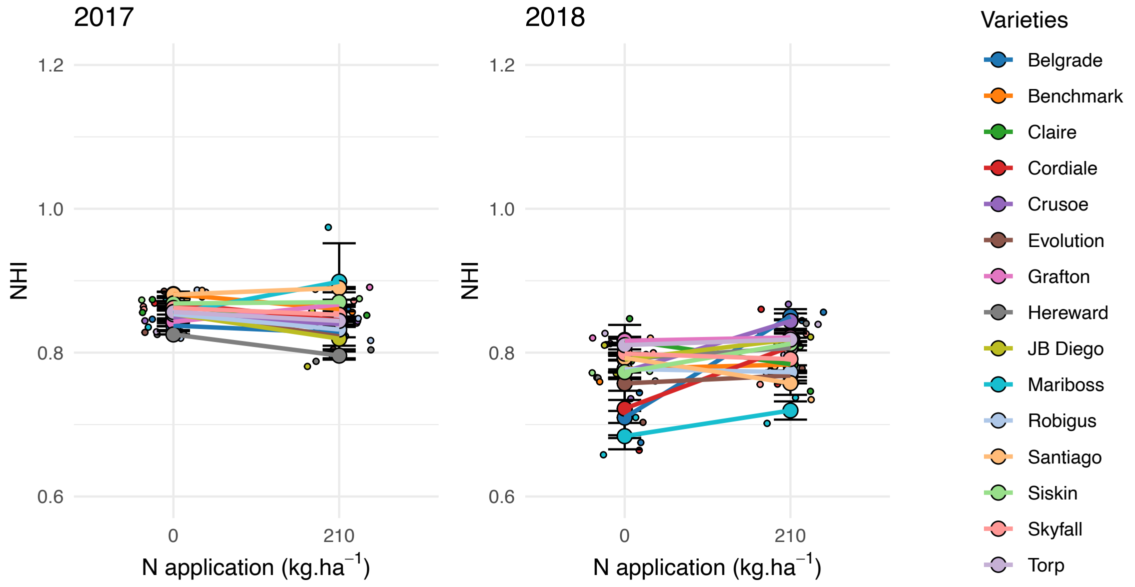

Figure S5: Steady increased in yield in more recently released varieties especially under higher N application rate. Data shown as BLUEs for yield and year of registration dates, as shown in Table 1.

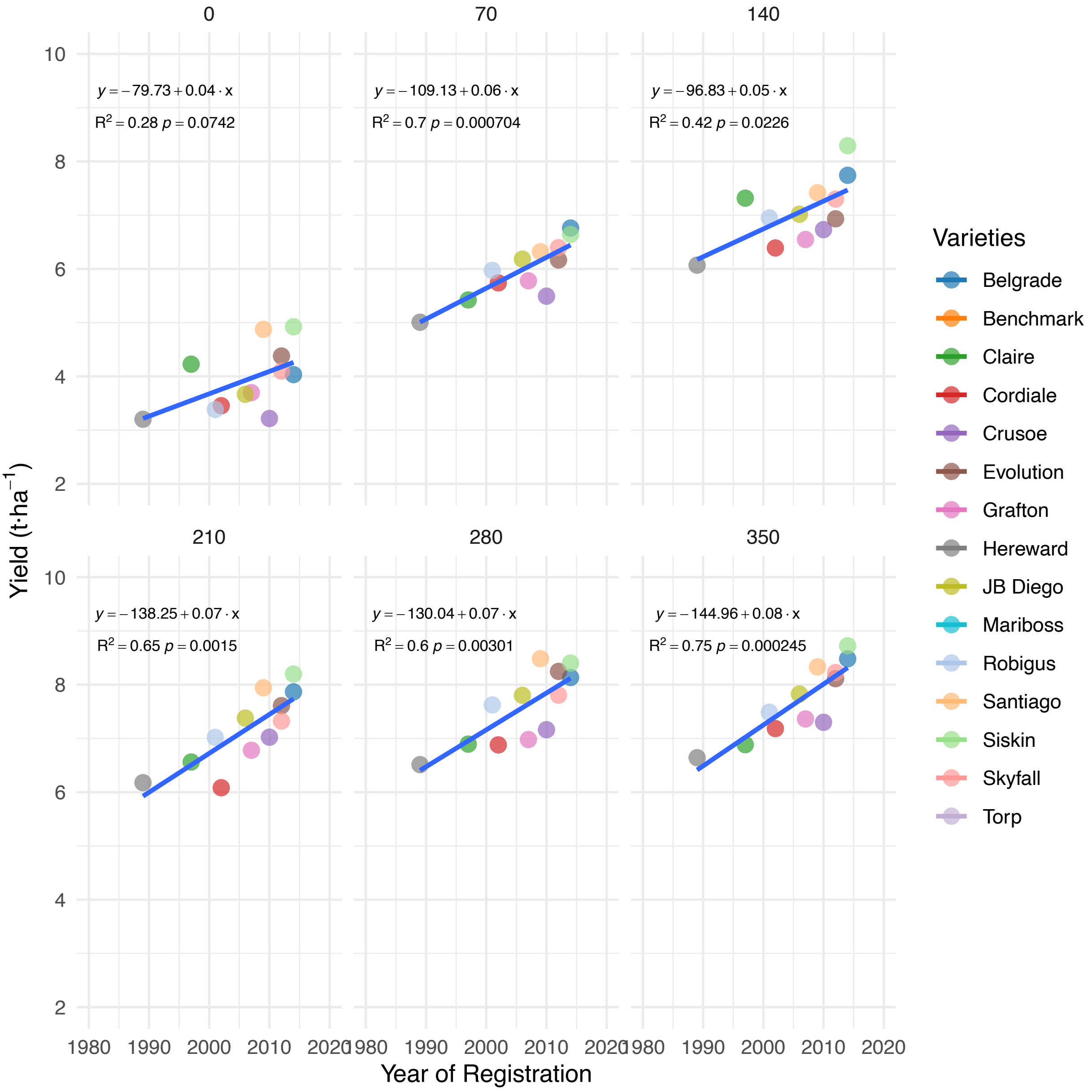

Figure S6: Varietal differences in the correlation between yield and NDVI at GS31.

Correlation between yield and NDVI measurements at GS31 for each individual variety.

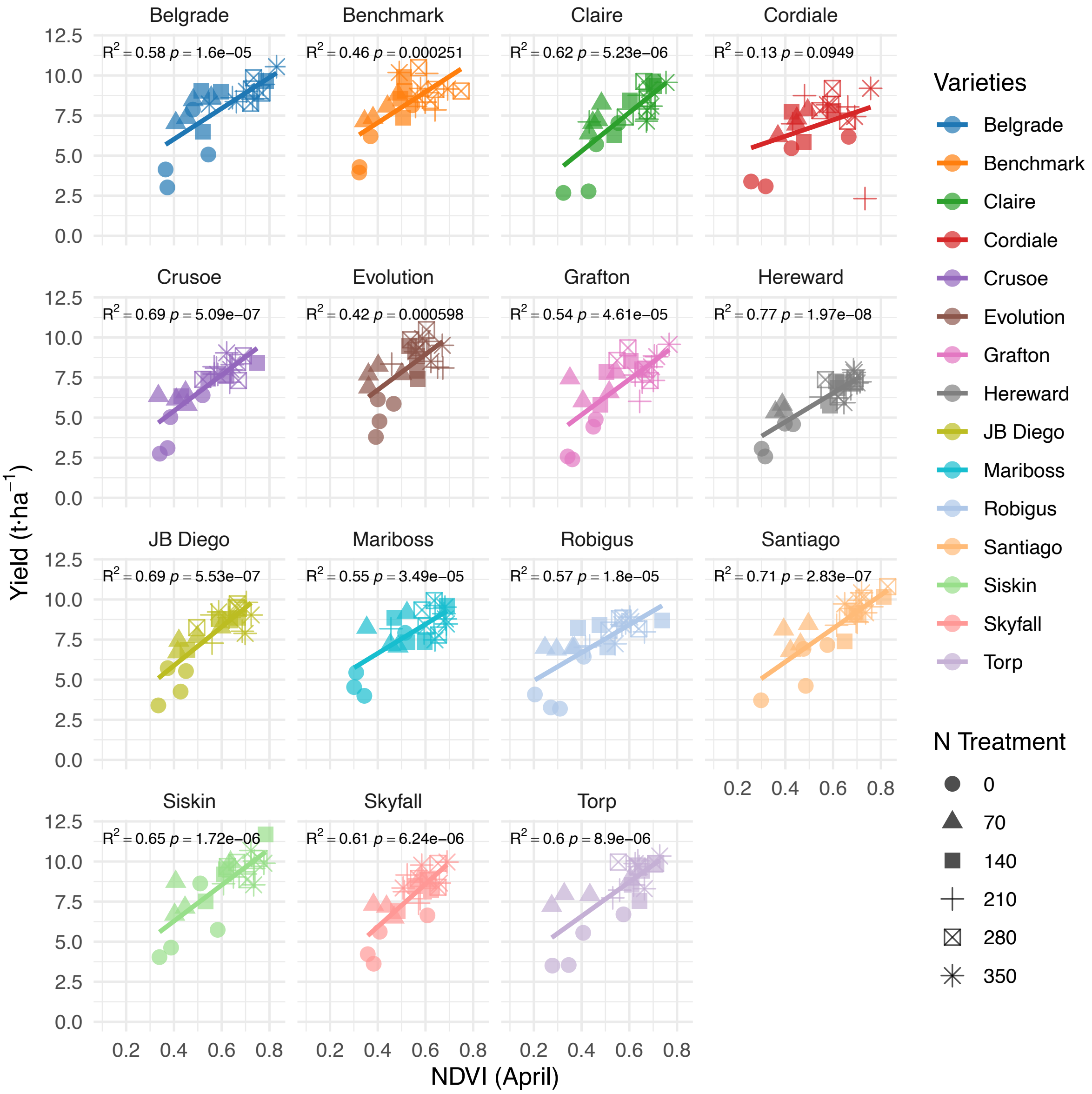

Figure S7: Higher correlation between NDVI and yield under lower N availability.
Correlation between yield and NDVI measurements at GS31 at each N rate.

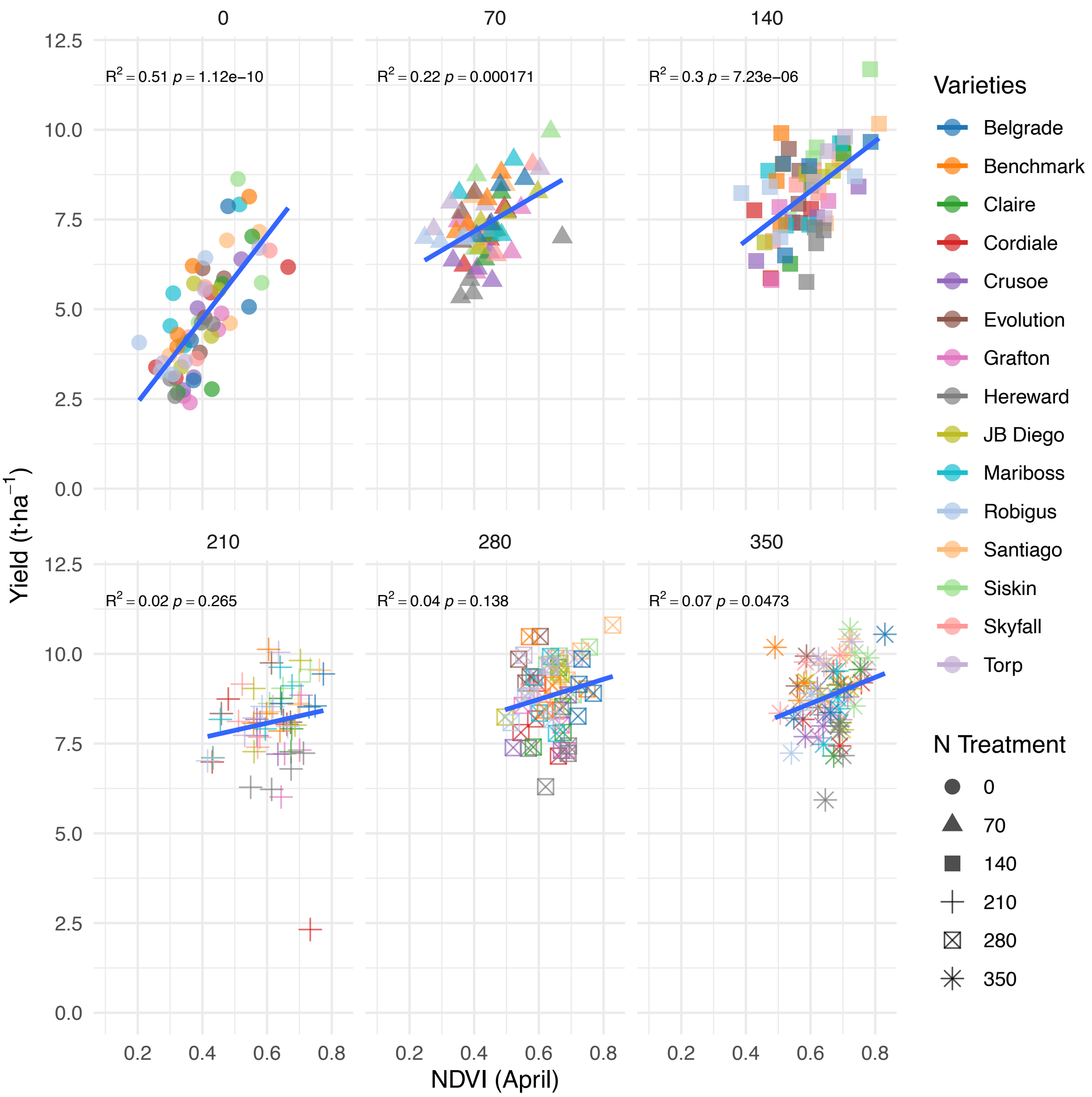

Figure S8: SPAD and LCC data correlate well over a range of N rate

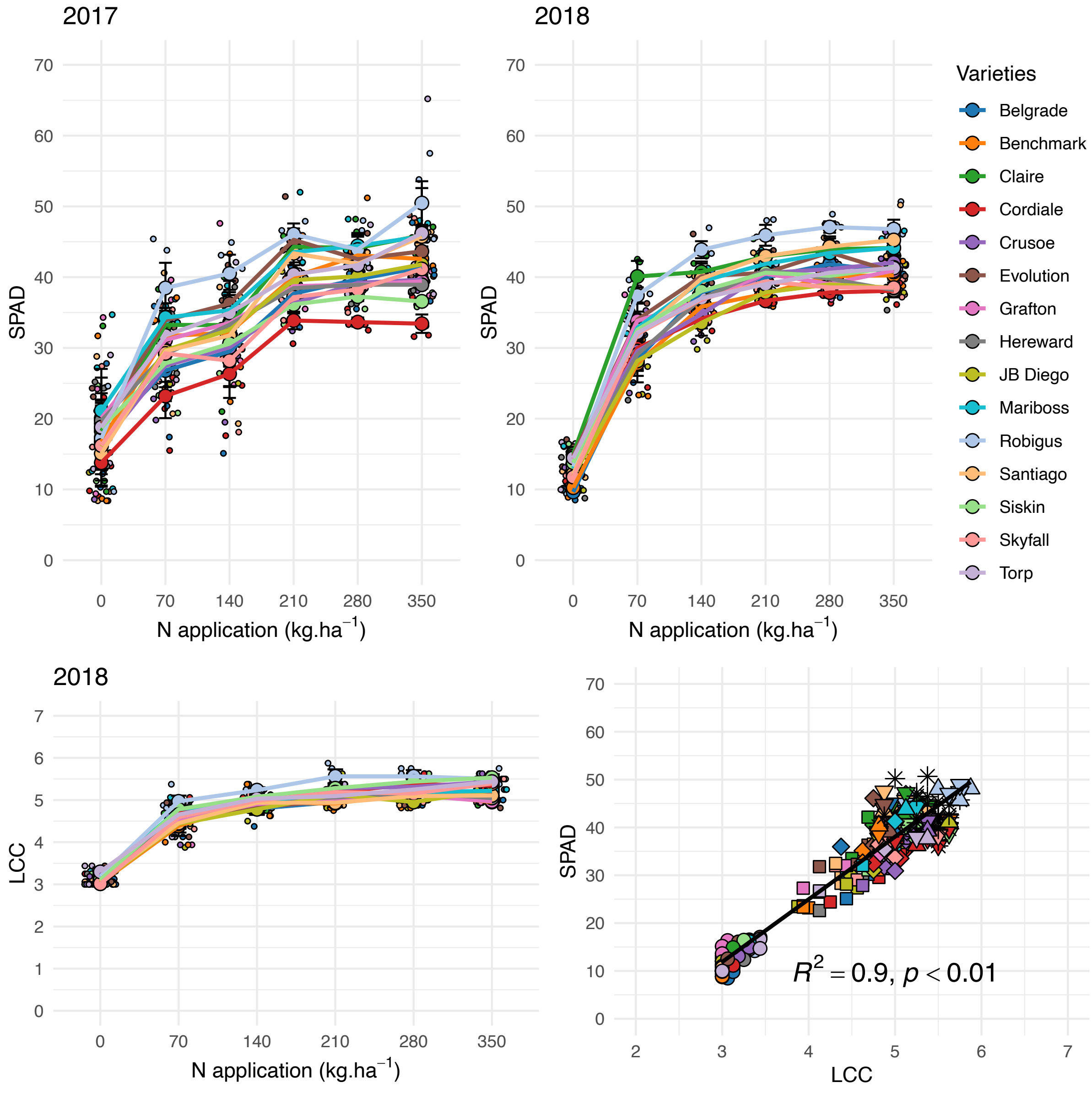

Figure S9: Varietal differences in spike weight Data shown as BLUEs from combined data for 2017 and 2018.

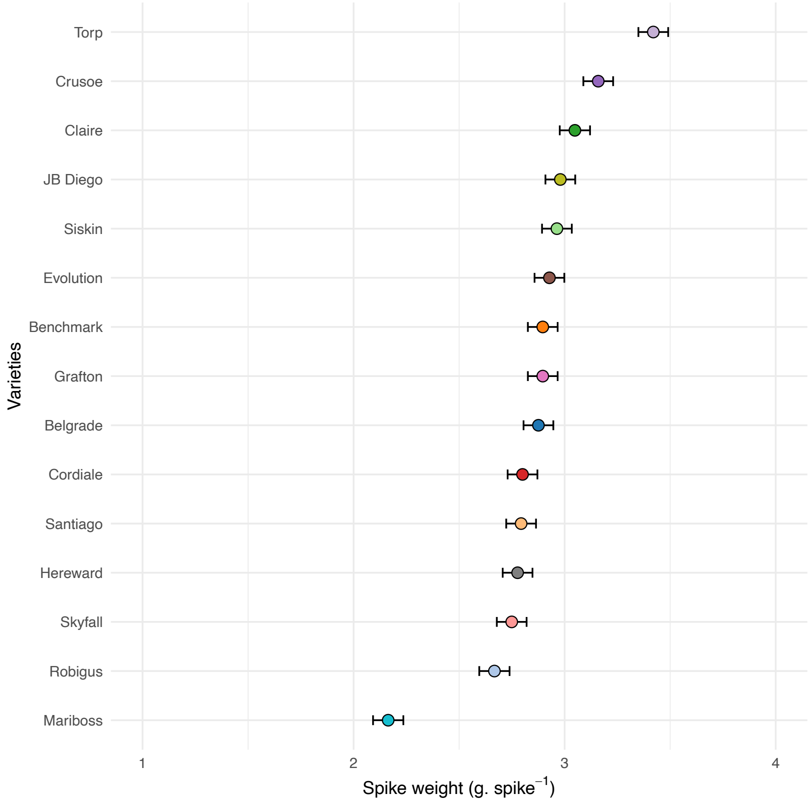

Figure S10: Significant differences in GPC amongst varieties Data shown as BLUEs from combined data for 2017 and 2018.

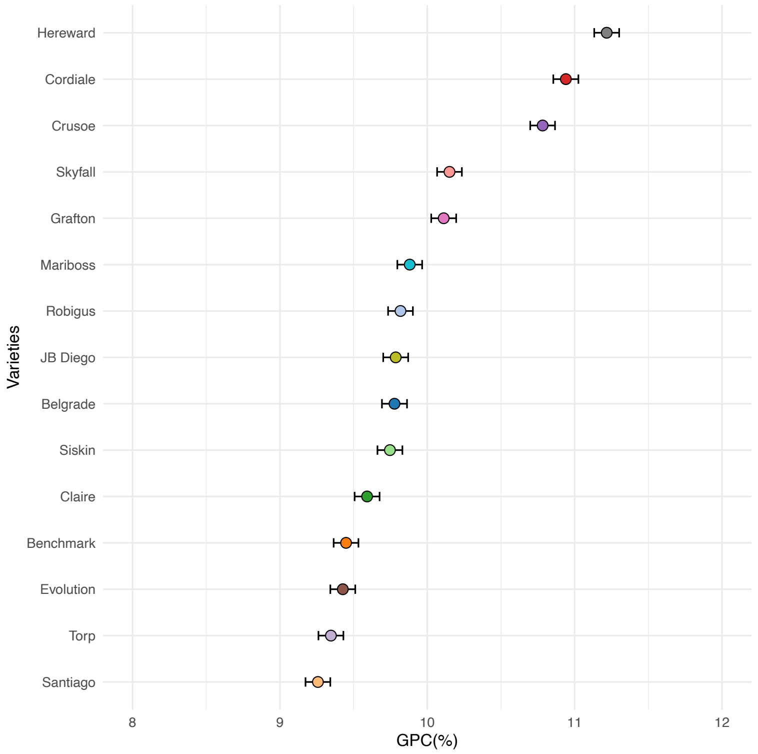

Figure S11: Correlation between GPC and Yield at different N rates. Data shown as individual datapoints and correlations are shown for each N levels.

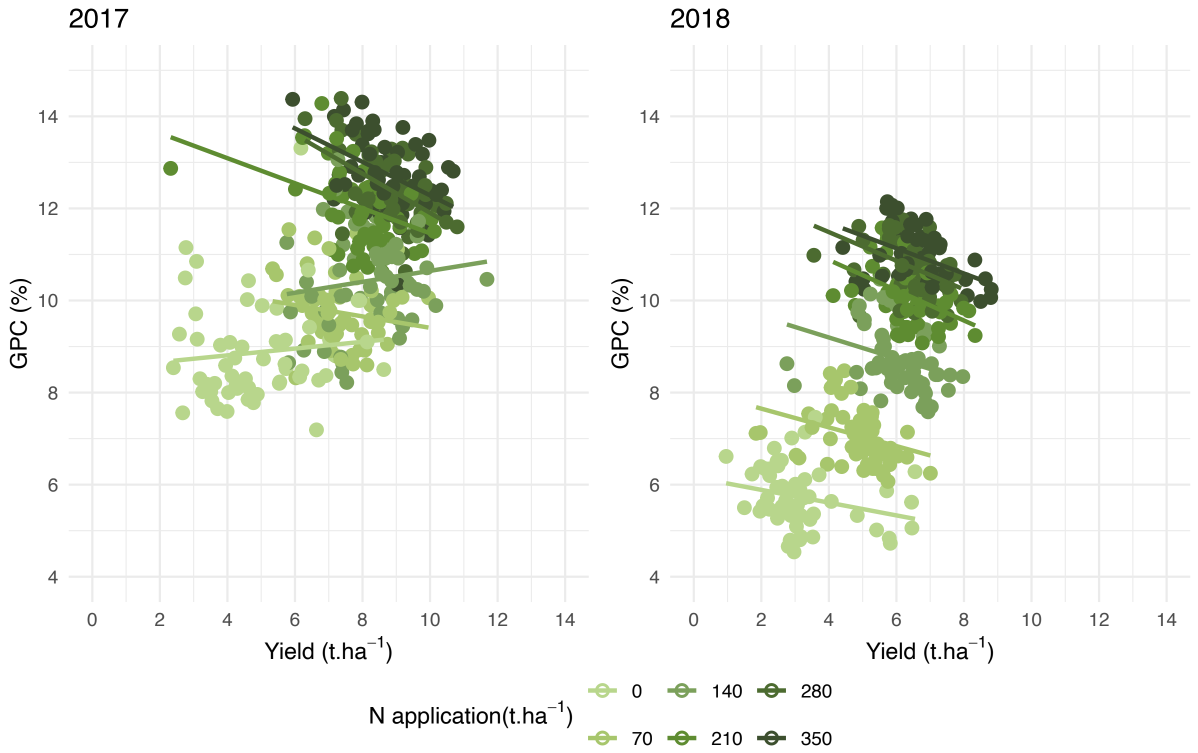

Figure S12: Phenology of each variety and under different N level in 2017. Data shown as individual plots, colour indicates N level with darker green indicating higher N level. Cordiale is shown as reaching GS31, GS55 and GS61 earlier than other varieties. Overall there was no measurable effect of N rate on phenology.

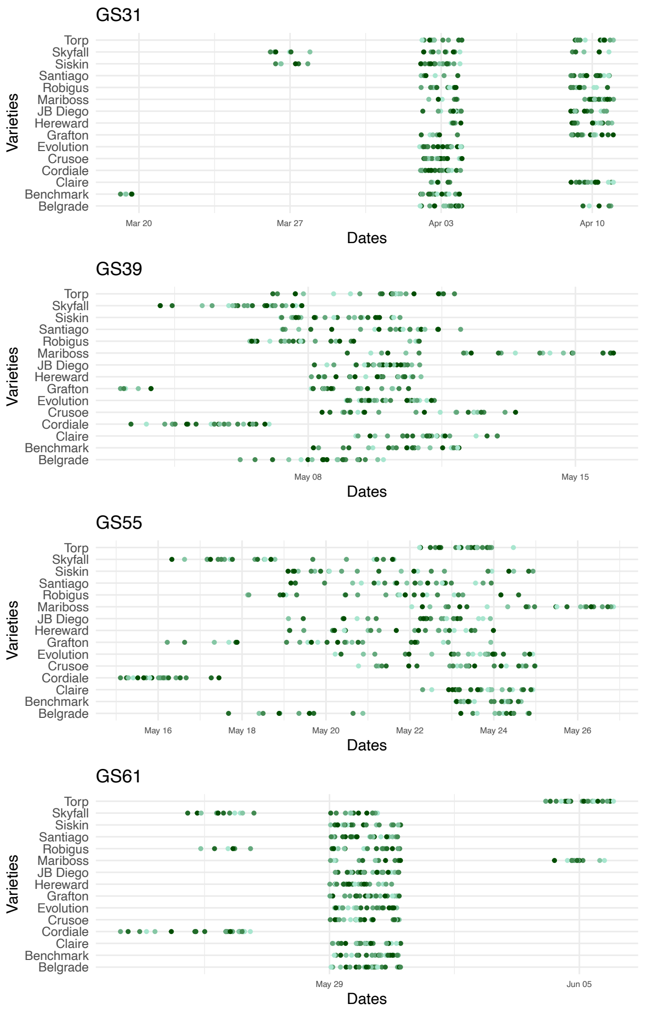

Supplement: Supplementary file 1 — Supplementary information [file 44264_2026_168_MOESM1_ESM.pdf]
